# Comparative effects of *Novirhabdovirus* genes on modulating constitutive transcription and innate antiviral responses, in different teleost host cell types

**DOI:** 10.1186/s12985-020-01372-4

**Published:** 2020-07-20

**Authors:** Bartolomeo Gorgoglione, Jeffery L. Ringiesn, Loc H. Pham, Brian S. Shepherd, Douglas W. Leaman

**Affiliations:** 1 Aquatic Animal Health Laboratory, Department of Pathobiology and Diagnostic Investigation, CVM & Department of Fisheries and Wildlife, CANR - Michigan State University, East Lansing, MI 48824 USA; 2 Department of Biological Sciences, Wright State University, 235 Diggs Laboratory / 134 Oelman Hall, 3640 Colonel Glenn Hwy, Dayton, OH 45435 USA; 3 Department of Biological Sciences, University of Toledo, 2801 W. Bancroft St, Toledo, OH 43606 USA; 4 USDA/ARS/School of Freshwater Sciences, University of Wisconsin-Milwaukee, 600 E. Greenfield Ave, Milwaukee, WI 53204 USA

**Keywords:** Virus-host interaction, Transfection, Pathobiology, Interferon, Salmonid, Viral pathogenesis, Cellular transcription, Fibroblastic cell, Epithelial cell, Viral hemorrhagic septicemia virus, Infectious hematopoietic necrosis virus

## Abstract

**Background:**

Infectious hematopoietic necrosis virus (IHNV) and viral hemorrhagic septicemia virus (VHSV) are highly contagious, pathogenic Novirhabdoviruses affecting fish and are thusly notifiable diseases with the World Organization for Animal Health. This study assessed the relative capacities of IHNV and VHSV genes to modulate host general transcription and explores the abilities of specific IHNV genes to interfere with the interferon pathway in heterogenous teleost cell-lines.

**Methods:**

Optimized protocols allowed for efficient transient transfections in EPC, BF-2, RTG-2 and RTgill-W1 cell lines of plasmids encoding IHNV (M genogroup) and VHSV (-IVb genotype) genes, including N, P, M, G and NV. Their impact on general cellular transcription was measured 48 hours post transfection (hpt) with luciferase constructs driven by a modified β-Actin promoter (pCAG). Their modulation of the innate antiviral immune response was characterized 72 hpt, using luciferase constructs measuring rainbow trout Type I IFN or MX-1 promoter augmentation, upon MAVS co-transfection.

**Results:**

M was generally confirmed as the strongest constitutive transcriptional suppressor while IHNV P, but not VHSV P, augmented constitutive transcription in fibroblastic cell types. Cell-specific effects were observed for viral G gene, with VHSV G exhibiting suppression of basal transcription in EPC and BF-2 but not in trout cells; while IHNV G was stimulatory in RTG-2, but inhibitory in RTgill-W1. NV consistently stimulated constitutive transcription, with higher augmentation patterns seen in fibroblastic compared to epithelial cells, and for IHNV NV compared to VHSV NV. The innate antiviral immune response, focusing on the IFN pathway, was silenced by IHNV M in all cell lines tested. IHNV N showed a dose-dependent suppression of type I IFN, but with minor effects on MX-1. IHNV P and G played minor IFN-inhibitory roles, consistent and dose-dependent only for G in rainbow trout cells. IHNV NV mediated a consistent stimulatory effect on either Type I IFN or MX-1, but much less pronounced in RTgill-W1.

**Conclusions:**

This study extends our understanding of Novirhabdoviruses-host interaction, showing differential innate immune responses in heterogenous cell types. Viral regulators of innate immune signaling are identified, either as dose-dependent suppressors (such as M and N) or stimulators (mainly NV), indicating novel targets for the design of more efficient vaccination strategies.

## Background

*Novirhabdovirus* genus includes two of the most important viruses affecting teleost fish and causing devastating epizootics to wild and farmed fish stocks in Europe, North America, and Asia [1, 2]. Viral hemorrhagic septicemia virus (VHSV), the *Piscine novirhabdovirus*, can infect over 90 species of marine and freshwater fishes across highly divergent teleost families [3–5]. VHSV is geographically distributed with 4 major genotypes and many sub-lineages and quasispecies strains [6]. Infectious hematopoietic necrosis virus (IHNV), the *Salmonid novirhabdovirus*, more specifically infects salmonid species [2]. IHNV is widespread with 5 major genogroups [7, 8] and is enzootic to western areas of North America [7, 9]. Even with different evolutionary dynamics, with IHNV evolving faster than VHSV [10], these viruses and their genogroups are co-circulating and often co-infecting hosts [10–14]. They also share a similar tropism, entering the host via epithelial surfaces, mainly through the gills and fins [15, 16]. Thereafter, they replicate in endothelial and hematopoietic tissues and induce similar symptomatology, characterized by hemorrhagic patterns [2, 17]. Novirhabdoviruses cause highly contagious and lethal diseases that are difficult to eradicate. Thus, both IHNV and VHSV are World Organization for Animal Health (OIE)-notifiable pathogens [4]. Despite eradication programs and decades of research to develop and test efficient control strategies [18, 19], the use of available vaccines is hampered by safety concerns and restrictions in commercial use [20, 21]. Comparative studies across viral types and hosts can enhance the ability to identify common features of virulence that may inform the development of targeted therapeutics. VHSV and IHNV share the same cohort of genes, but the diversity of VHSV strains isolated and sequenced vastly outnumbers available information on IHNV, and the host responses to pathogen challenge vary widely. Given the presence of VHSV-IVb throughout the Great Lakes watershed there is tangible risk to all farmed fish in the region [22, 23]. VHSV-IVa/b are reported to cause mild to modest disease in rainbow trout (*Oncorhynchus mykiss*) [24, 25], but disease kinetics and mortalities may be comparable to those seen in yellow perch (*Perca flavescens*) and round gobies (*Neogobius melanostomus*), which are highly susceptible to this pathogen [26–28]. Additional challenge studies show that VHSV-IVb can cause significant morbidity and mortality in rainbow trout [29, 30]. Given differences in the reported susceptibility of trout to VHSV-IVb, the documented rapid evolution of this pathogen in the Great Lakes may allow for adaptation towards increased virulence in rainbow trout, that could parallel what occurred with IHNV [7, 23, 31]. This could enable the disease to go undetected in a production setting. Therefore, although VHSV is not considered an immediate threat to trout aquaculture industry in North America, it is critical to understand the infectious capacity of VHSV-IVb in rainbow trout in parallel with IHNV, as a means to dissect the host-virus interaction during *Novirhabdovirus* pathobiology. Thus, comparative immunogenicity assessments can shed light on host- or strain-specific mechanistic differences with useful indications for therapeutic purposes.

Novirhabdoviruses are bullet-shaped enveloped viruses, with non-segmented negative-sense single-stranded RNA genomes of 11,131 bases for IHNV [32] and 11,158 bases for VHSV [33]. Six open reading frames, separated by conserved gene junctions, encode for 5 structural and one non-structural components: Nucleocapsid protein (N), polymerase-associated Phosphoprotein (P), Matrix protein (M), surface Glycoprotein (G), NonVirion (NV) and a large RNA polymerase (L). The Matrix gene is known as the most powerful inducer of apoptotic changes, inhibiting the host-directed gene expression by blocking nascent cellular RNA synthesis, thus efficiently suppressing host antiviral responses [34, 35]. The transmembrane G protein, key for virus entry and recognition, has been the main target for vaccine development, possessing major antigenic properties [36–38]. The NV gene is unique in Novirhabdoviruses, distinguishing them from Rhabdoviruses of non-fish hosts, and genetically diverging between them [39]. NV protein’s precise functions remain unknown. NV is a small, non-structural protein [12 and 14 kDa, respectively in IHNV and VHSV], shown to be essential for viral growth [40], replication [41] and pathogenicity [42]. Previous work suggested that NV might play a role in suppressing host IFN-1 and MX-1 through inhibiting NF-κB activity [41, 43, 44]. Salmonids can mount a complex antiviral response, with either secreted or intracellular Type I IFN orchestrating interferon-stimulated gene (ISG) transcription at early infection stages [45–47]. Type I IFN transcripts strongly correlate with viral burden and with the transcription of marker genes encoding for effectors of the IFN antiviral cascade [48, 49]. The sustained expression of IGSs, viz MX proteins, ISG-15 or VIPERIN, is a common hallmark adopted in fish immunology as a measure of the antiviral state induced upon viral infections [50–54], and heterogeneous co-infections [55, 56]. As such, a potential role for NV in suppressing host innate immunity would represent a novel function unique to *Novirhabdovirus*.

The aims of this study were to utilize specific reporter plasmids to characterize the impact of individual IHNV genes on constitutive transcription on innate antiviral transcriptional responses. Transfection protocols were improved, achieving adequate transient transfection efficiency in EPC, BF-2, RTG-2 and RTgill-W1 cell lines, to compare results between two epithelial (EPC and RTgill-W1) and two fibroblastic (BF-2 and RTG-2) cell types. Comparative studies on the actions of *Novirhabdovirus* proteins will enable identification of specific anti-host activities of these proteins in varying host cell-lines, with the aim of identifying genetic viral regulators that interfere with general cell transcription, or selectively with the host innate immunity signaling. Such information could identify key viral factors to be targeted for development of more efficient vaccines to combat these pathogens.

## Methods

### Cell cultures

Epithelial and fibroblastic cell lines were retrieved from American Type Culture Collection (ATCC), including: Fathead minnow (*Pimephales promelas*) *Epithelioma papulosum cyprini* (EPC) (ATCC: CRL-2872); Bluegill (*Lepomis macrochirus*) fry (BF-2) (ATCC: CCL-91); Rainbow trout (*Oncorhynchus mykiss*) gonad (RTG-2) (ATCC: CCL-55); and Rainbow trout gill (RTgill-W1) (ATCC: CRL-2523). Cell cultures were maintained in 25 cm^2^ tissue culture flasks (CytoOne) at 20 °C, with L-15 Leibovitz media (HyClone) supplemented respectively with 1% Penicillin-Streptomycin (PS) solution (Corning), and with 2% (L15-2PS) or 10% (L15–10PS) fetal bovine serum (Corning). Before using, media were filtered through a 0.2 μm cellulose nitrate membrane (Nalgene). Confluent cell monolayers were split 1:2 or 1:3 to seed 5 × 10^5^ cells to each well of the 12-wells plate (CytoOne) in L15–10PS and grown for 72 h in standard conditions before each transfection experiment.

### Plasmids and luciferase reporters

To investigate the transcriptional modulatory effects of selected IHNV proteins, including N, P, M, G (structural) and NV (non-structural), specific genes from M genogroup were cloned into expression plasmids for transient co-transfections experiments. Only the L gene, encoding for the RNA-dependent RNA polymerase, was not included in this screening due to its much larger size (respectively of 6091 nt in IHNV and 5954 nt in VHSV). Target coding sequences were PCR-amplified using cDNA from archive viral stocks with specific primers (Table 1). PCR fragments were cloned into EcoRI and KpnI sites of pcDNA3.1(−)Myc/His A plasmid (Invitrogen). Plasmids were amplified in *E. coli* DH5α cells and plasmid DNA (pDNA) was purified using PureLink Fast Low-Endotoxin Midi Plasmid Purification Kit™ (Invitrogen), following the manufacturer’s instructions. All expression plasmids were confirmed by sequencing before use in transfection experiments. The construction of other expression plasmids was previously reported [35]. Luciferase reporter constructs, harboring *Renilla reniformis* luciferase gene under the transcriptional control of the promoter of each testing gene, were reported previously, including: simian virus 40 early promoter (SV40)/luc, Type I IFN/luc and MX-1/luc [35, 57, 58]. The CMV enhancer/chicken β-Actin promoter, pCAG/luc plasmid [59], was purchased from Addgene (Plasmid #55764).

**Table 1:** Oligonucleotides used for the construction of plasmids

Primer	Sequence (5′ → 3′)	Restriction Site	Primer Source	Sequence Source
IHNV-M N se	AG**GAATTC**ATGACAGCGACACTCAGAG	EcoRI		HM461966 (AEH95651)
IHNV-M N as	AG**GGTACC**GTGGAATGAGTCGGAGTC	KpnI		
IHNV-M P se	AG**GAATTC**ATGTCGATGGAGAAGGAG	EcoRI		HM461966 (AEH95652)
IHNV-M P as	AG**GGTACC**TTGACTTGCTTCATGCGC	KpnI		
IHNV-M M se	AC**GAATTC**ATGTCTATTTTCAAGAGAGC	EcoRI	Ke et al.*,* 2017 [35]	HM461966 (AEH95653)
IHNV-M M as	CTT**GGTACC**TTTTTCCTTCCCCCGCTTTTCGG	KpnI		
IHNV-M G se	A**GAATTC**GAGATGGACCATGATCACCAC	EcoRI		HM461966 (AEH95654)
IHNV-M G as	A**GGTACC**TTGGACCGGTTTGCCAGGTG	KpnI		
IHNV-M NV se	AC**GAATTC**ATGGACCACCGCGACATAAACAC	EcoRI		HM461966 (AEH95655)
IHNV-M NV as	AC**GGTACC**TCTGGGATAAGCAAGAAAGTCTTC	KpnI		
VHSV-IVb N se	CA**GAATTC**ATGGAAGGAGGAATC	EcoRI	Ke et al.*,* 2017 [35]	KY359357 (ASZ84902)
VHSV-IVb N as	GT**GGTACC**ATCAGAGTCCTCG	KpnI		
VHSV-IVb P se	CA**GAATTC**ATGACTGATATTGAGAT	EcoRI	Ke et al.*,* 2017 [35]	KY359357 (ASZ84903)
VHSV-IVb P as	GT**GGTACC**CTCTAACTTGTCCA	KpnI		
VHSV-IVb M se	AC**GAATTC**ATGGCTCTATTCAAAAGAAAGCGCACCATCCTG	EcoRI	Ke et al.*,* 2017 [35]	KY359357 (ASZ84904)
VHSV-IVb M as	AC**GGTACC**CCGGGGTCGGACAGAG	KpnI		
VHSV-IVb G se	AC**GAATTG**ATGGAATGGAATACTT	EcoRI	Ke et al.*,* 2017 [35]	KY359357 (ASZ84905)
VHSV-IVb G as	GT**GGTACC**GACCATCTGGCT	KpnI		
VHSV-IVb NV se	AC**GAATTC**ATGACGACCCAGTCGGCAC	EcoRI	Ke et al.*,* 2017 [35]	KY359357 (ASZ84906)
VHSV-IVb NV as	AC**GGTACC**TGGGGGAGATTCGGAGCCA	KpnI		

The restriction enzyme recognition sites are shown in bold. Sequence source is provided for both the full viral genome and for specific genes (in parenthesis)

### Transient cell transfection

Cell transfection was performed in L15–10, without addition of antibiotics, using the suitable transfection reagent for each cell line at a final 3:1 reagent volume to total DNA ratio. EPC and BF-2 cells were transfected using FuGENE™ HD Transfection Reagent (Promega), while RTgill-W1 and RTG-2 using ViaFect™ Transfection Reagent (Promega). Plasmid concentrations in all transfection experiments were equalized between samples by the inclusion of closed circular empty vector pcDNA3.1, which was also used in negative control groups. DNA mixtures were complexed with the respective transfection reagents in 37 °C pre-warmed Opti-MEM™ I reduced serum medium (Gibco), then incubated for 15 min at 37 °C. 100 μl transfection doses were added to each confluent cell monolayer with 500 μl L15–10 in each well. 12-well plates (Greiner Bio-One) were incubated at 20 °C, without any further medium replacement or manipulation until the indicated sampling points.

### Cell viability assay

Cell viability was quantified by staining cell monolayers with Sulforhodamine B (SRB) (Invitrogen) [60]. Cells seeded in 96-well plates (Greiner Bio-One) were transfected as described above, thereafter fixed with 10% (w/v) trichloroacetic acid solution for 15 min and stained with 0.4% (w/v) SRB/1% (v/v) acetic acid solution for 20 min. Plates were washed four times with 1% (v/v) acetic acid and dried at RT. Dye was eluted in 10 mM unbuffered Tris-Base by incubating on a shaker for 5 min at RT. Absorbance was read using a microplate reader (Synergy H1, BioTek) at 550 nm, with values averaged between replicates. Cell viability was assessed 48 h after viral gene transfection using variable plasmid concentrations, thus excluding cytotoxic effects due to the transient transfection processes (Supp. Fig. 1).

### Immunoblotting

The expression of viral plasmids transfected in fish cells was assessed by Western blotting. Cell lysate prepared and separated by SDS-polyacrylamide gel electrophoresis (PAGE), as previously described [61]. Samples were electrophoretically transferred to Immobilon^®^-P PVDF membrane (MilliporeSigma) and membranes were blocked with 5% (w/v) BSA/TBST (P-753, Boston Bioproducts) for 1 h at RT. Primary antibodies, anti-Myc monoclonal antibody (Myc, Invitrogen) and anti-β-Actin (Sigma) were diluted in TBST at 1:5000 and incubated overnight at 4 °C. Membranes were incubated with the secondary antibody for 1 h at RT, using horseradish peroxidase (HRP)-conjugated Goat anti-mouse IgG1 (Invitrogen) at 1:10,000 dilution in TBST. Immunoreactive bands were visualized with SuperSignal™ West Pico PLUS chemiluminescent substrate (Thermo Scientific) using Amersham Imager 600 (General Electric). Following exposure with anti-Myc antibodies, membranes were stripped using Restore™ PLUS western blot stripping buffer (Thermo Scientific) for 10 min at RT, thereafter, blocked with 5% (w/v) BSA/TBST for 30 min at RT, and re-probed with the anti β-Actin antibody.

### Luciferase reporter assay

After the designated time post-transfection, cell monolayers in each well were gently washed with 1X PBS at 20 °C, then lysed for 15 min at RT in 120 μl of luciferase cell culture lysis reagent (Promega), used at 1.5X in molecular grade water (HyClone). Sampled 12-well plates were kept at −20 °C until assays were performed. From each cell lysate sample, 75 μl was collected for Luciferase reporter assay in 96-well white solid flat bottom opaque microplates (Greiner Bio-One), and 10 μl for Bradford assay in 96-well clear flat bottom microplates (Greiner Bio-One). The luciferase reporter assay was performed by adding to each sample 100 μl of a mixture containing: 51 μl of Luciferase assay ATP assay buffer [3.83 mM EGTA (MP Biomedicals), 14.4 mM Magnesium sulphate (Fisher Chemical), 23.9 mM Glycylglycine (ICN Biomedicals), 14.4 mM Potassium phosphate dibasic (Fisher BioReagents), 0.98 mM DTT (Dithiothreitol, Fisher BioReagents), 1.97 mM ATP (MP Biomedicals), 0.33 mM Coenzyme-A (MP Biomedicals), in Milli-Q water], and 49 μl of Luciferin solution [1 mM DTT, 25.1 mM Glycylglycine, 0.27 mM D-Luciferin (Pierce), in autoclaved Milli-Q water]. Luminescence light emission was measured with a microplate reader (Synergy H1, BioTek), setting top optics reading and luminescence spectral scanning gain/sensitivity to 135. The total protein load was measured by adding 10 μl of each cell lysate to 90 μl of Pierce™ Coomassie Plus (Bradford) solution (diluted 50% in autoclaved Milli-Q water). Light absorbance values were immediately read at 595 nm with a Synergy H1 microplate reader.

### Data analysis

Luminescence data, expressed as Relative Light Units (RLU), were normalized to lysate protein concentrations. The Relative Luciferase Activity (RLA) was calculated as the % ratio between stimulated (co-transfected with testing plasmids) and unstimulated (pcDNA3.1 alone) samples. RLA ratio was analyzed using one-way ANOVA and LSD post hoc test for comparison of group means. Statistical analyses were performed and graphically represented using GraphPad Prism version 6 (GraphPad Software Inc.). All data shown are representatives of at least three independent experiments and presented as group means (±SEM). Changes relative to the control sample were considered statistically significant when *p* < 0.05.

### Experimental design

#### Constitutive cellular transcription experiments

The impact of single IHNV genes on host constitutive transcription was initially measured using a constitutively active SV40-luciferase reporter plasmid construct. This approach aimed to corroborate results from a previous assessment of VHSV genes in EPC cells [35]. Fish cell lines EPC, BF-2, RTG-2 and RTgill-W1, were co-transfected with SV40/luc and two doses of each IHNV gene (Supp Fig. 3). The endpoint for this experiment was set at 48 hpt, coincident with the time post-infection when IHNV and VHSV begin to induce morphological changes in infected cells, including initial cytopathic effects [37, 62]. To confirm and extend these observations, the approach was repeated with a different reporter construct, pCAG-luciferase (pCAG/luc). The pCAG promoter is a hybrid CMV/β-Actin promoter that yields high-level constitutive expression in different cell lines [63]. The experimental set-up was unchanged, with pDNA amounts experimentally optimized for each cell line, and assessment performed with both VHSV and IHNV plasmid constructs.

#### Antiviral response modulation experiments

To assess the impact of single IHNV genes on the host innate immune response we targeted two sequential stages of the IFN response pathway. The first approach was to measure the modulation of Type I IFN promoter activity. IHNV genes were co-transfected in fish cell lines, together with a luciferase construct regulated by the rainbow trout Type I IFN-a promoter (IFN/luc), along with MAVS, co-transfected to activate the IFN promoter [57, 64]. Fish cell lines were co-transfected with IFN/luc and MAVS along with two doses of each IHNV plasmid gene construct. pDNA amounts were again experimentally optimized for each cell line.

IFNs signal through conserved JAK/STAT pathways to upregulate the expression of ISGs, thus playing a crucial role in the innate immune response to *Novirhabdovirus* infection [45, 47]. To measure ISG induction, the MX-1 promoter was used as a marker for assessing IFN activation in the presence or absence of IHNV genes. Cell lines were co-transfected with MX-1/luc and MAVS, and with two doses of each IHNV gene. pDNA construct amounts were experimentally optimized for each cell line (specified in Fig. 4) to achieve readable RLU from all cell lines. IFN and MX-1 experiments proceeded for 72 hpt, to provide enough time for MAVS to efficiently stimulate the IFN pathway and its downstream effectors.

## Results

Studies aimed at identifying the roles of VHSV and IHNV genes on host responses were carried out in several fish cell lines. The use of two commercial reagents allowed us to optimize transfection efficiency for the cell lines used, allowing for reproducibility between comparative experiments performed in parallel. Transfection efficiency was optimized by directly adding FuGENE HD (Promega)- or ViaFect (Promega)-DNA mixtures (doses prepared in OptiMEM) to each cell monolayer (in fresh L15–10 medium, without PS). Preliminary studies identified FuGENE HD as the best reagent for EPC and BF-2, while ViaFect as optimal for the RTG-2 and RTgill-W1 cell-lines, in terms of readable RLU values obtained and lack of cytotoxicity for cell monolayers upon transfection. The most reproducible results were obtained transfecting confluent monolayers at 72 h post seeding (incubating cells at 20 °C in standard conditions). The expression of each INHV and VHSV gene was checked at 48 hpt by Western Blotting (Supp. Fig. 2). All pDNA doses were experimentally determined for each cell type and used throughout these studies; see each figure legend for specific amounts.

### Comparative analysis of constitutive transcription modulation

Analysis of constitutive transcriptional modulation by individual IHNV genes was initially performed using SV40/luc as a reporter plasmid in transiently transfected teleost cell lines. IHNV N elicited a slight dose-dependent downregulation of luciferase activity in both epithelial cell lines (Supp Fig. 3A and B). Interestingly, the opposite trend was observed in fibroblastic cells, including a mild stimulatory effect in BF-2, but a strong dose-dependent induction in RTG-2 (Supp Fig. 3C and D). IHNV P and IHNV G mediated modest effects or had no impact on SV40/luc expression in the cell lines tested. IHNV M protein consistently inhibited general transcription in all cell lines tested. IHNV NV generally augmented expression, particularly in EPC (Supp Fig. 3A) and BF-2 (Supp Fig. 3C). NV stimulatory effects were less marked in both rainbow trout cell lines, although data from the latter experiments require careful interpretation: RLU values obtained upon SV40/luc transient transfection were strongly elevated in EPC cells (~ 75,000 RLU in control) and BF-2 cells (~ 2000 RLU in control), but RTgill-W1 (~ 1200 RLU in control) and RTG-2 (~ 200 RLU in control) barely rose above background. Because of the poor activity of SV40/luc in rainbow trout cells, we investigated the use of another constitutively active promoter for analysis (see below).

**Fig. 1:**
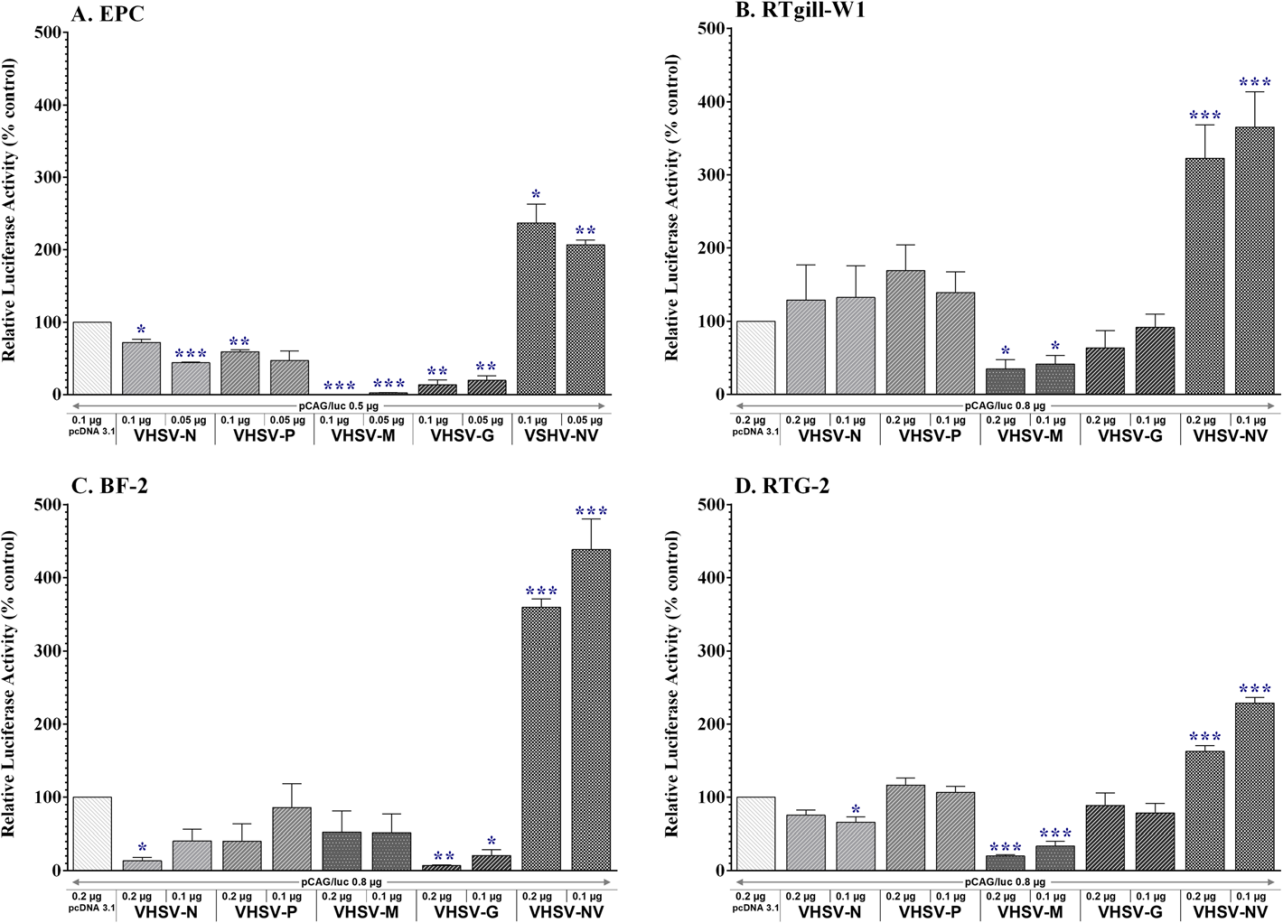
Comparative modulation of host constitutive transcription by single VHSV genes. Epithelial (**a** EPC; **b** RTgill-W1) and fibroblastic (**c** BF-2; **d** RTG-2) cell lines were co-transfected with pCAG/luc plus two doses of each VHSV gene plasmid. Closed circular empty pcDNA3.1 plasmid vector was used for transfection balancing and baseline control. Luciferase activity was analyzed at 48 hpt and RLU normalized to total protein concentration in each sample. Data are representative of three independent experiments. Values are group means ±SEM. *p < 0.05; **p < 0.01; ***p < 0.001 indicate significant differences from pcDNA control values as determined by one-way ANOVA and Fisher’s LSD test

**Fig. 2:**
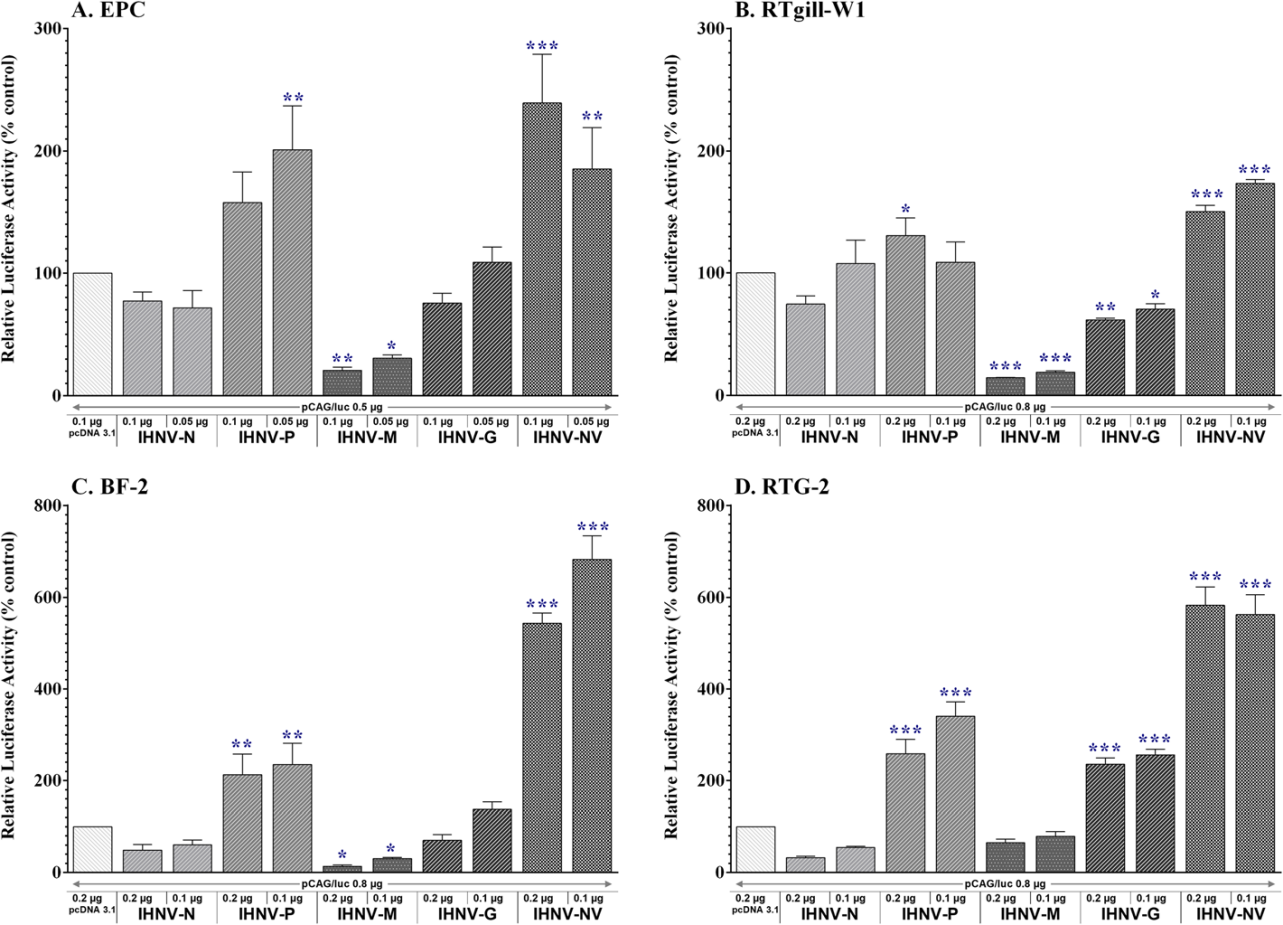
Comparative modulation of host constitutive transcription by single IHNV genes**.** Epithelial (**a** EPC; **b** RTgill-W1) and fibroblastic (**c** BF-2; **d** RTG-2) cell lines were co-transfected with pCAG/luc plus two doses of each IHNV gene plasmid. Closed circular empty pcDNA3.1 plasmid vector was used for transfection balancing and baseline control. Luciferase activity was analyzed at 48 hpt and RLU normalized to total protein concentration in each sample. Data are representative of three independent experiments. Values are group means ±SEM. *p < 0.05; **p < 0.01; ***p < 0.001 indicate significant differences from pcDNA control values as determined by one-way ANOVA and Fisher’s LSD test

In contrast to the limitations observed with SV40/luc, a modified β-Actin promoter luciferase reporter plasmid (pCAG/luc) gave reliably higher and more consistent RLU values in all the fish cell lines tested, thus providing improved sensitivity of host constitutive transcriptional regulation. RLU values obtained upon pCAG/luc transient transfection were much higher in EPC (~ 330,000 RLU in control), BF-2 and RTG-2 (~ 30,000 RLU in control), and lower but still much improved in the RTgill-W1 cell line (~ 16,000 RLU in control). Experiments were therefore performed as in Supp Fig. 3, but this time using pCAG/luc to better determine how VHSV and IHNV genes impacted constitutive expression in heterogeneous fish cell lines. With few exceptions, M from both viruses significantly inhibited pCAG/luc expression in all cells (Figs. 1 and 2). In the few instances where VHSV or IHNV M effects were not statistically significant (in BF-2 and RTG-2, respectively) the trend was toward reduced expression as compared with controls. In contrast, NV from both viruses significantly augmented pCAG/luc expression, particularly in BF-2 where augmentation was as much as 6 to 7 fold over control values (Figs. 1 and 2). The other genes exhibited variable effects that were sometimes contradictory across cells or viruses of origin. VHSV P, for example, only rarely impacted luciferase expression to a significant extent (in BF-2, and at only one dose; Fig. 1), whereas IHNV P augmented pCAG/luc expression in all cell types at one or both doses tested (Fig. 2). Similarly, VHSV G suppressed luciferase expression in the non-rainbow trout cells but had no effect in RTG-2 and RTgill-W1 (Fig. 1). IHNV G had the opposite impact, augmenting pCAG/luc expression in the rainbow trout cell lines (Fig. 2b, d), but it had no effect in EPC or BF-2 cells (Fig. 2a, b). These data highlight ways in which genes from these related rhabdoviruses can differentially impact transcription in different host backgrounds.

### Modulation of type I IFN transcription in teleost cell lines

**Fig. 3:**
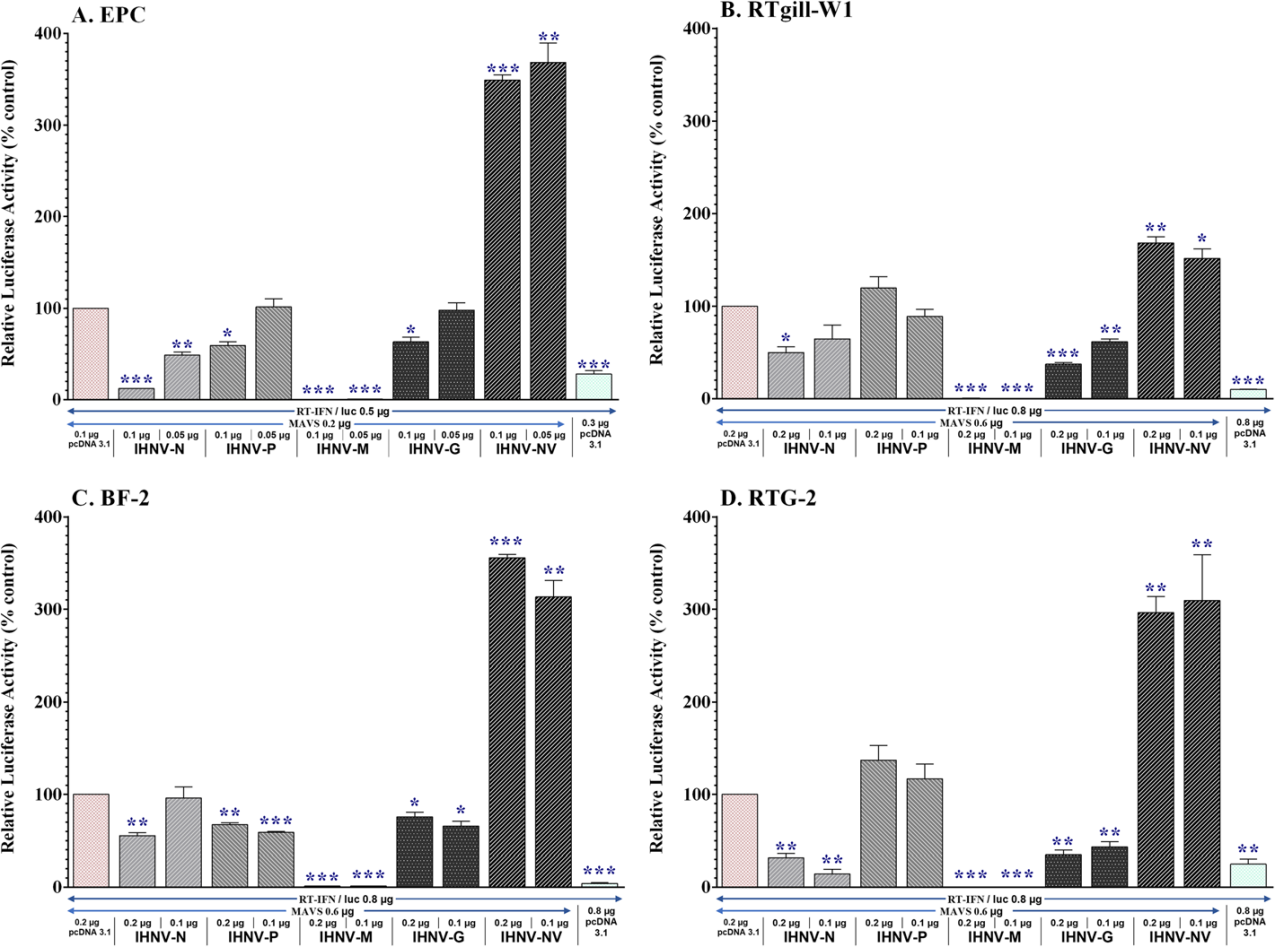
Comparative modulation of host innate antiviral response by single IHNV genes. Epithelial (**a** EPC; **b** RTgill-W1) and fibroblastic (**c** BF-2; **d** RTG-2) cell lines were co-transfected with rainbow trout Type I IFN/luc, with MAVS as a basal IFN expression stimulator, plus two doses of each IHNV gene plasmid. Closed circular empty pcDNA3.1 plasmid vector was used for transfection balancing and baseline control. Luciferase activity was analyzed at 72 hpt and RLU normalized to total protein concentration in each sample. Data are representative of three independent experiments. Values are group means ±SEM. *p < 0.05; **p < 0.01; ***p < 0.001 indicate significant differences from pcDNA control values as determined by one-way ANOVA and Fisher’s LSD test

Our previous studies had implicated VHSV genes in the modulation of Type I IFN regulatory pathways in EPC cells [35]. To determine whether IHNV genes similarly impacted IFN gene transcription, each of the fish cell lines was transfected with a rainbow trout IFN promoter/luciferase construct (RT-IFN/luc) along with MAVS to upregulate its expression. MAVS, alone, upregulated RT-IFN/luc by 3 to 20 fold, depending on the cell type, and in all cases IHNV NV co-transfection augmented RT-IFN induction by an additional 2 to 4 fold (Fig. 3). M, on the other hand, suppressed RT-IFN/luc expression to background levels in all cells at both doses tested, indicating a more potent transcriptional suppressive effect as compared to its impact on pCAG/luc, particularly in RTG-2 cells (compare to Fig. 2). Interestingly, IHNV G exhibited a slight but significant suppression of RT-IFN/luc in all cells at one or both doses assessed, but P was more selective, inhibiting the pathway only in the non-rainbow trout cells (Fig. 3a, c). IHNV N exhibited the most clearly differential effects on IFN transcription, as compared to its effects on constitutive transcription. In all cells tested, IHNV N inhibited IFN promoter expression at one or both doses, as compared to having no effect on the pCAG/luc plasmid (compare Figs. 3 to 2). IHNV P only modestly altered the expression of RT-IFN/luc in EPC and BF-2.

### Comparative analysis of ISG transcription modulation

**Fig. 4:**
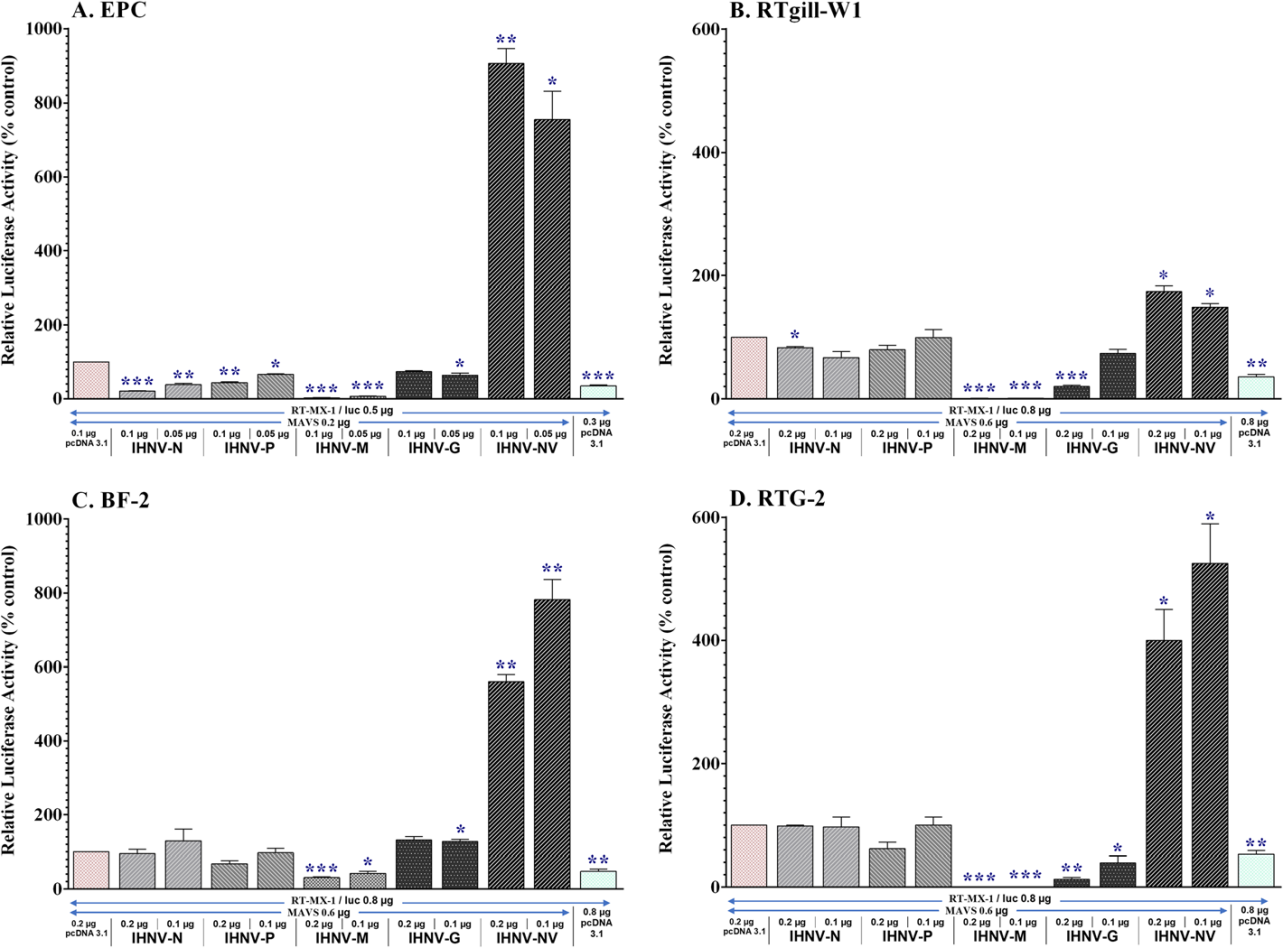
Comparative modulation of host innate antiviral response by single IHNV genes. Epithelial (**a** EPC; **b** RTgill-W1) and fibroblastic (**c** BF-2; **d** RTG-2) cell lines were co-transfected with rainbow trout MX-1/luc, with MAVS as a basal IFN expression stimulator, plus two doses of each IHNV gene plasmid. Closed circular empty pcDNA3.1 plasmid vector was used for transfection balancing and baseline control. Luciferase activity was analyzed at 72 hpt and RLU normalized to total protein concentration in each sample. Data are representative of three independent experiments. Values are group means ±SEM. *p < 0.05; **p < 0.01; ***p < 0.001 indicate significant differences from pcDNA control values as determined by one-way ANOVA and Fisher’s LSD test

Once produced, IFNs upregulate the expression of hundreds of effector genes, generally referred to as ISGs. To assess the impact of IHNV genes on ISG regulation, IFNs were activated by MAVS transfection as in Fig. 3, and then downstream ISG regulation assessed using the RT-MX1/luc reporter plasmid. MX-1 is potently regulated by IFNs and was transcriptionally activated in these studies by using MAVS co-transfection to upregulate endogenous IFN. The two strongest effects observed were with IHNV M and NV co-transfection with the RT-MX-1/luc plasmid (Fig. 4). As with the RT-IFN/luc plasmid, IHNV M potently suppressed transcription from the induced RT-MX-1/luc plasmid, while IHNV NV potently augmented luciferase activity in three of the four cells types (4 to 9 fold), and less robustly but still significantly in the fourth (2 fold; Fig. 4). Few other genes produced dramatic effects, although both P and N suppressed MX-1 promoter activity in EPC cells. The action of IHNV NV and VHSV NV plasmids was further confirmed by disrupting the coding sequences upon restriction enzyme cleavage (using Kpn1/EcoRI), which resulted in the evident loss of any stimulatory activity on MX-1 (Supp Fig. 4). Overall, these data suggest that the IFN response was less susceptible to IHNV proteins, except for NV and M.

## Discussion

Successful viral infection and shedding relies on evolutionarily conserved strategies to selectively counteract innate immune defenses in susceptible host species. In many instances, one or more viral proteins must block or attenuate specific cellular pathways to allow efficient virus replication and to propagate the infection. Differential effects of *Novirhabdovirus* genes on general cellular transcription, or targeted transcription of antiviral effector genes, were investigated in this study using a comparative approach involving use of different teleost cell lines. By measuring differences in viral protein alteration of host transcriptional responses across epithelial (EPC and RTgill-W1) and fibroblastic cell types (RTG-2 and BF-2), representing different fish species, we sought to identify functional differences that might correlate with host transcriptional responses to each virus species. We focused on Novirhabdoviruses of economic or ecological importance in North America: VHSV-IVb and IHNV-M genotypes. VHSV-IVb is of significant ecological concern in the Laurentian Great Lakes region where sporadic outbreaks have occurred over the past 17 years [31, 65, 66], thus there is tangible risk to all farmed fish in the region. But to date, VHSV-IVb has shown limited pathogenicity toward rainbow and brown trout [24, 54], this could enable the disease to go undetected in a production setting. In contrast, IHNV is highly pathogenic to many salmonid species, inducing potent innate immune response including upregulation of Type I IFN and MX-1 [67] and leading to outbreaks of significant economic consequence in the trout aquaculture industry [2, 68]. Cell line susceptibility is generally consistent with the disease susceptibility of originating host sources [69], with VHSV-IVa being more pathogenic than VHSV-IVb towards RTgill-W1 cells [70].

By testing the effects of four of the major structural (N, P, M, G), and the sole nonstructural (NV), genes from VHSV-IVb and IHNV-M in a series of transient transfection studies, we were able to gain insight into the comparative efficacy of each genes ability to modulate general or innate immune gene transcription in different teleost host backgrounds. Our data suggest that the effects of the *Novirhabdovirus* M and NV proteins remain the most robust modulators of host response measures in the teleost cell lines used in this study. Beyond these two genes, the potential roles of other viral components have not been thoroughly assessed across a range of host cells using optimized transfection-plasmid construct procedures. As such, we feel that these studies add to our understanding of host-virus interactions in this important family of fish viruses and pave the way forward for further comparative studies using optimized in vitro procedures.

As mentioned, previous work had focused on M protein’s anti-host actions. Long known to perturb transcription [34], recent studies have focused on the mechanism of action of the *Novirhabdovirus* M gene, confirming that VHSV M potently suppressed transcription in a manner reminiscent of M from mammalian Rhabdoviruses [35, 71]. The studies also demonstrated that M protein’s viral packaging function could be separated genetically from its anti-host actions [35], similar to vesicular stomatitis virus (VSV) M protein [72]. Consistent with the above body of literature, M significantly reduced host transcription in most cells tested and under most conditions in our studies. Although some variability was observed across cell types tested, which is as expected, the most consistent effects were obtained with VHSV and IHNV M proteins. Interestingly, M from these two related novirhabdoviruses elicited similar effects on transcription across different host types. M from both IHNV and VHSV-IVb suppressed both constitutive transcription and transcription associated with the Type I IFN pathway. Although a few experiments failed to discern a significant impact of M on transcription (VHSV M in BF-2 and IHNV M in RTG-2), these were associated more with the post-transfection sampling time point (e.g. 48 hpt) than the biology, since later time points routinely showed potent suppression (data not shown). Although the observed transcriptional inhibition by VHSV and IHNV M were predicted based on prior reports, these new data demonstrate the broad spectrum of conditions under which the effect can be observed, including both natural and unnatural hosts (salmonid and non-salmonid cell lines).

Rhabdoviral G protein is a critical determinant of cellular engagement, immunogenicity and in some cases host specificity [20, 73–75]. In this study, VHSV G protein had no effect on constitutive transcription within epithelial (RTgill-W1) and fibroblastic (RTG-2) rainbow trout cell lines. IHNV G, in contrast, showed mild suppression of constitutive, and innate immune regulated, transcription in gill epithelial cells but strongly stimulatory in gonad fibroblastic cells. Furthermore, IHNV G induced dose-dependent inhibition to either Type I IFN and MX-1, more marked in both rainbow trout cell lines. G uniquely interferes with multiple host cell functions, including translation through the endoplasmic reticulum, which may activate negative feedback loops that impact IFN responses over the 72 hpt studies reported herein. This action looks indeed less marked on the general transcription at 48 hpt, and in EPC and BF-2 cells transfected with rainbow trout-specific IFN pathway reporters, but the explanation for this remains unclear at this point in time.

The novirhabdoviral N gene is a determinant for virulence variability [76, 77]. N is implicated in the innate immune response activation, by cytotoxic T cells specifically recognizing N-derived peptides presented in MHC class I [75]. The development of experimental vaccines with rearranged gene order showed that the position of IHNV N plays the most critical role in determining the level of viral attenuation [78]. VHSV N did not modulate the IFN pathway in EPC cells at 48 hpt [35]. In our studies at 72 hpt, the impact of N was dependent not only on the viral source, but also on the reporter gene used. IHNV N suppressed the IFN promoter-luciferase reporter construct (Fig. 3) but had minimal effects on the MX-1 promoter (Fig. 4). This interesting distinction between Type I IFN and MX-1 might lead to future work on those aspects of the innate immune response that are impacted by the novirhabdoviral N protein.

The P gene is another viral component critical for virus replication, and in some mammalian rhabdoviruses the P protein is implicated in the modulation of innate immune signaling, particularly through IRF3 phosphorylation [79]. N and P genes have been implicated in determining virulence of VHSV genotypes/strains in rainbow trout [77]. In this study, P had no consistent effects on the assessed signaling pathways in the cell lines examined. IHNV P positively regulated the constitutive pCAG/luc plasmid at one or more doses in all cells tested, but more consistently in fibroblastic cell types (Fig. 2). In contrast, IHNV P either had no effect, or inhibited only slightly the other reporter constructs. Our previous studies had failed to identify an impact of VHSV P in EPC cells using a wide array of reporter constructs [35]. It thus seems that fish rhabdoviruses may either be distinct from their mammalian counterparts, or that we simply have not identified an appropriate gene (reporter construct), cell line, or timeframe to monitor for anti-host activity of P in teleost cell lines.

While the functions of IHNV and VHSV structural genes in viral replication are generally well characterized, the function of the NV gene is still not fully understood. Previous studies identified NV protein anti-apoptotic activity [80]. NV nuclear localization in RTG-2 cells was deemed necessary for optimal IHNV growth and pathogenicity [43]. NV may support viral replication through inhibition of the IFN system, based on work in that same report [43]. rVHSV-ΔNV was highly attenuated in EPC cells [81] as compared to wild type rVHSV. Reverse genetics with interactomic analysis recently identified PPM1Bb (a member of the PP2C family of Ser/Thr protein phosphatases) as a cellular partner of the VHSV NV protein. PPM1Bb recruitment by NV induces a strong inhibition of both RIG-I- and TBK1-mediated IRF-3-dependent IFN and ISG promoter activities [82]. Overall, however, no uniform picture of NV function has yet emerged.

In all our studies, NV consistently augmented luciferase expression (host transcription) for both VHSV and IHNV (~ 2 to ~ 8 fold-increases; Figs. 1 and 2, Supp Fig. 3). IHNV NV was particularly robust in inducing the IFN pathway in four heterogeneous teleost cell lines. An IHNV NV plasmid in which the coding sequence was excised by restriction enzyme cleavage (Kpn1/EcoRI) resulted in the loss of any stimulatory activity (Supp Fig. 4). It is well-known that Novirhabdoviruses are highly susceptible to Type I IFN responses [83, 84], and so this novel NV effect requires further investigation. Additionally, a recent study has also provided evidence that VHSV NV plays a role in the mediation of the PKR-like endoplasmic reticulum kinase (PERK)-eukaryotic initiation factor 2α (eIF2α) pathway through increased levels of phosphorylated eIF2α and viral-mediated host translational shutoff, leading to efficient viral protein synthesis and decreased IFN production during infection [61]. It appears that the conserved abilities of M protein to broadly suppress host transcription, and NV to stimulate antiviral responses, should be considered as a potential coordination nexus for teleost *Novirhabdovirus* pathobiology. Their combined action could give the virus enough time for replication, followed by release of mature viral particles. Further study is needed to describe NV immune-enhancing features and the relative activities across different hosts and viral strains.

The available literature dealing with the impact of viral gene components on host transcription often rely on sub-optimal transfection methods and conditions. The development of optimized transfection protocols for fish cell lines, notoriously difficult to transfect, is an important challenge in the field, and limits basic research on teleost virology and related diagnostic applications. The availability of new transfection reagents allowed the selection for the best method to retrieve robust and comparable data from heterogenous cell types, using consistent treatment conditions and post-transfection time points. Newer generation commercial reagents, including ViaFect and a new version of Fugene, have drastically increased the transfection efficiency for fish cell lines. Importantly, when paired with the use of a stabilized cell culture medium (L-15, with a stable pH and without CO_2_ supplementation), these newer transfection reagents were used under identical conditions allowing the use of a more simplified and consistent set of conditions. Cell monolayers can be maintained under consistent conditions and receive minimal manipulation as compared to prior methods. Our efforts enabled refinement of experimental conditions, including timing for transfecting cells post plating and the optimal dose of plasmids being used. Together, these validated transfection protocols augmented our ability to answer comparative questions on the function of *Novirhabdovirus* genes.

## Conclusions

This study analyzed the relative individual abilities of *Novirhabdovirus* genes to interfere with general host cell transcription and demonstrated the modulatory effects of IHNV genes the Type I IFN pathway in various teleost cell lines. A consistent transcriptional inhibitory action was observed for M, with less robust suppression observed for N. NV exhibited a novel consistent stimulatory effect on constitutive transcription and antiviral immunity across a variety of cell types. Other viral genes studied, P and G, showed some more specific induction patterns, linked to the cell type or plasmid dose. This study provides novel insights on the viral regulators of the innate signaling, thus suggesting further studies are required to discover the mechanistic interaction eliciting the stimulatory effect seen for NV. In support of this, the comparative approach used in this study allowed a glimpse into a more comprehensive picture of the novirhabdoviral pathogenetic strategy across a variety of cells and teleost hosts. This was enabled by optimization of multiple cell transfection methods, treatment times and reporter genes tested. These results underscore the value of comparative in vitro immunological assessments involving use of more than a single virus or host cell type/line and optimized transfection techniques. Results from this study will be helpful in identifying novel viral targets, such as NV, that modulate the host-pathogen interaction and immunogenicity, thus informing more efficient vaccination and vaccine development strategies.

## Supplementary information

**Additional file 1: Supp. Fig. 1.** Confirmation of cell viability after transient transfection. EPC cells (5 × 10^3^) were grown for 72 h and thereafter transfected with various concentrations of plasmids encoding IHNV genes. Closed circular empty pcDNA3.1 plasmid vector was used for transfection balancing and baseline control. At 48 h post transfection sulforhodamine B (SRB) viability assays were performed to determine the cytotoxicity of the overexpressed viral proteins. pcDNA sample values were normalized to an additional non-transfected average value; thus, all viral gene samples were further normalized to the new pcDNA value, creating a relative viability. Data were plotted as an average with standard deviation (*N* = 4).

**Additional file 2: Supp. Fig. 2.** Confirmation of transient transfection in EPC cells. EPC cells (1 × 10^6^) were grown for 72 h and thereafter transfected with 2 μg of each plasmids, respectively encoding for IHNV (**a**) or VHSV (**b**) genes in frame with a C-terminal Myc epitope tag. Cell transfection was achieved using FuGENE™ HD transfection reagent (Promega) in Opti-MEM™ I (Gibco). A non-transfected (NT) sample was included for control. The closed circular empty pcDNA3.1 plasmid vector was used for transfection balancing and for negative control. Cell lysates were sampled at 48 h post transfection and separated by SDS-PAGE and immunoblotted for protein expression with an anti-Myc antibody. Afterwards, blots were stripped and re-probed with an anti-β-Actin antibody to show loading controls.

**Additional file3: Supp. Fig. 3.** Comparative modulation of host constitutive transcription by single IHNV genes. Epithelial (**a** EPC; **b** RTgill-W1) and fibroblastic (**c** BF-2; **d** RTG-2) cell lines were co-transfected with SV40/luc plus two doses of each IHNV gene plasmid. Closed circular empty pcDNA3.1 plasmid vector was used for transfection balancing and baseline control. Luciferase activity was analyzed at 48 hpt and RLU normalized to total protein concentration in each sample. Data are representative of three independent experiments. Values are group means ±SEM. *p < 0.05; **p < 0.01; ***p < 0.001 indicate significant differences from pcDNA control values as determined by one-way ANOVA and Fisher’s LSD test.

**Additional file 4: Supp. Fig. 4.** Confirmation of Novirhabdoviruses modulation of the host innate antiviral response operated by NV gene. Epithelial (**a** EPC) and fibroblastic (**b** BF-2) cell lines were co-transfected with rainbow trout MX-1/luc, with MAVS as a basal IFN expression stimulator, plus 0.1 μg of intact of destroyed IHNV or VHSV NV gene plasmid. NV plasmids were destroyed upon restriction enzyme cleavage (using Kpn1/EcoRI). Closed circular empty pcDNA3.1 plasmid vector was used for transfection balancing and baseline control. Luciferase activity was analyzed at 72 hpt and RLU normalized to total protein concentration in each sample. Data are representative of three independent experiments. Values are group means ±SEM. *p < 0.05; **p < 0.01; ***p < 0.001 indicate significant differences from pcDNA control values as determined by one-way ANOVA and Fisher’s LSD test.

## Data Availability

All data generated or analyzed during this study are included in this published article, and its supplementary information files.
