# Supplementary figures and images for: Comparative effects of Novirhabdovirus genes on modulating constitutive transcription and innate antiviral responses, in different teleost host cell types
Source: Virol J. 2020 Jul 20;17:110. doi: 10.1186/s12985-020-01372-4 (PMC7369537; doi:10.1186/s12985-020-01372-4)

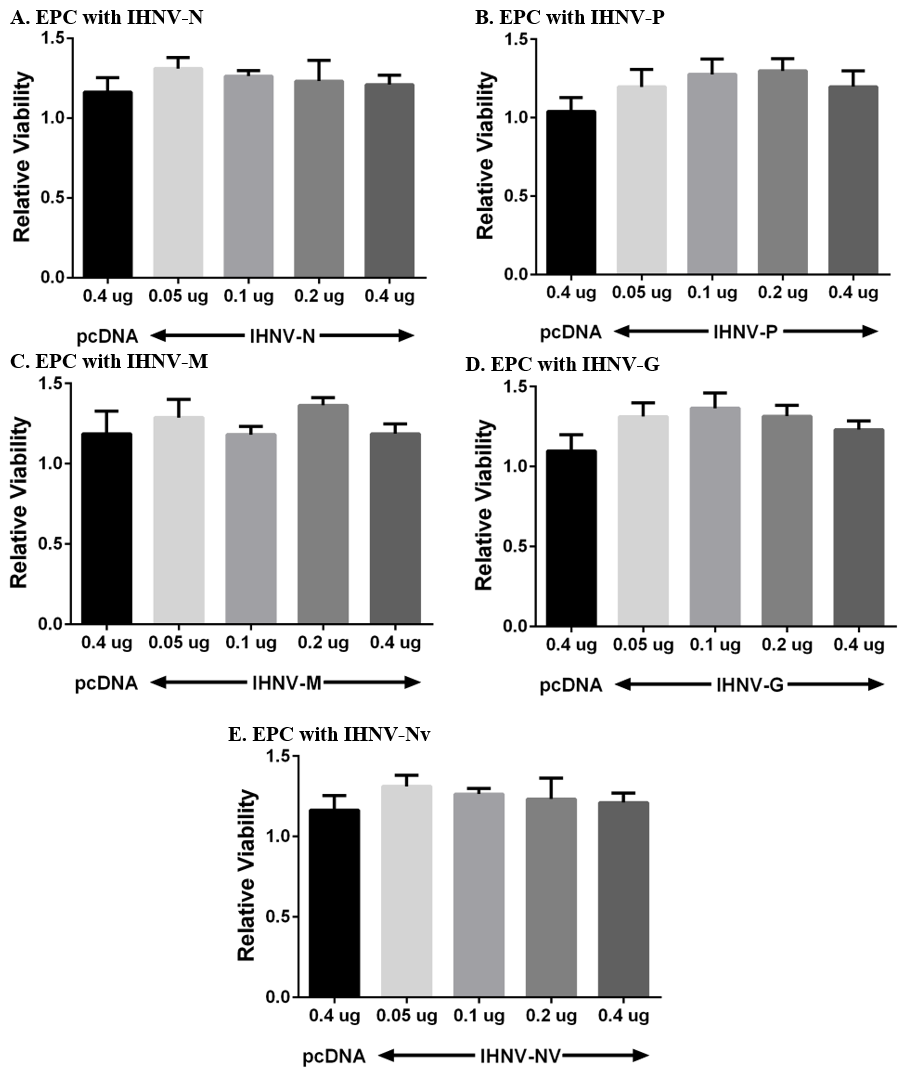

Supplement: Supplementary file 1 — Additional file 1: Supp. Fig. 1. Confirmation of cell viability after transient transfection. EPC cells (5 × 10^3^) were grown for 72 h and thereafter transfected with various concentrations of plasmids encoding IHNV genes. Closed circular empty pcDNA3.1 plasmid vector was used for transfection balancing and baseline control. At 48 h post transfection sulforhodamine B (SRB) viability assays were performed to determine the cytotoxicity of the overexpressed viral proteins. pcDNA sample values were normalized to an additional non-transfected average value; thus, all viral gene samples were further normalized to the new pcDNA value, creating a relative viability. Data were plotted as an average with standard deviation (N = 4). [file 12985_2020_1372_MOESM1_ESM.tif]

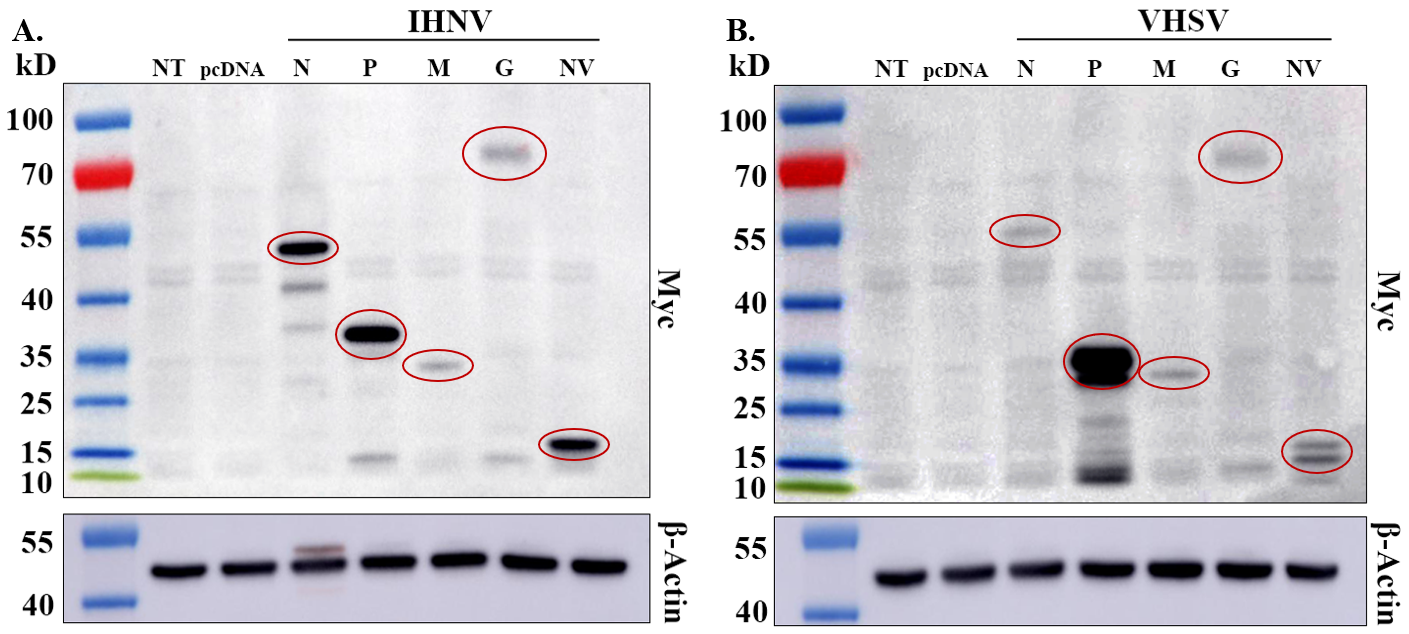

Supplement: Supplementary file 2 — Additional file 2: Supp. Fig. 2. Confirmation of transient transfection in EPC cells. EPC cells (1 × 10^6^) were grown for 72 h and thereafter transfected with 2 μg of each plasmids, respectively encoding for IHNV (a) or VHSV (b) genes in frame with a C-terminal Myc epitope tag. Cell transfection was achieved using FuGENE™ HD transfection reagent (Promega) in Opti-MEM™ I (Gibco). A non-transfected (NT) sample was included for control. The closed circular empty pcDNA3.1 plasmid vector was used for transfection balancing and for negative control. Cell lysates were sampled at 48 h post transfection and separated by SDS-PAGE and immunoblotted for protein expression with an anti-Myc antibody. Afterwards, blots were stripped and re-probed with an anti-β-Actin antibody to show loading controls. [file 12985_2020_1372_MOESM2_ESM.tif]

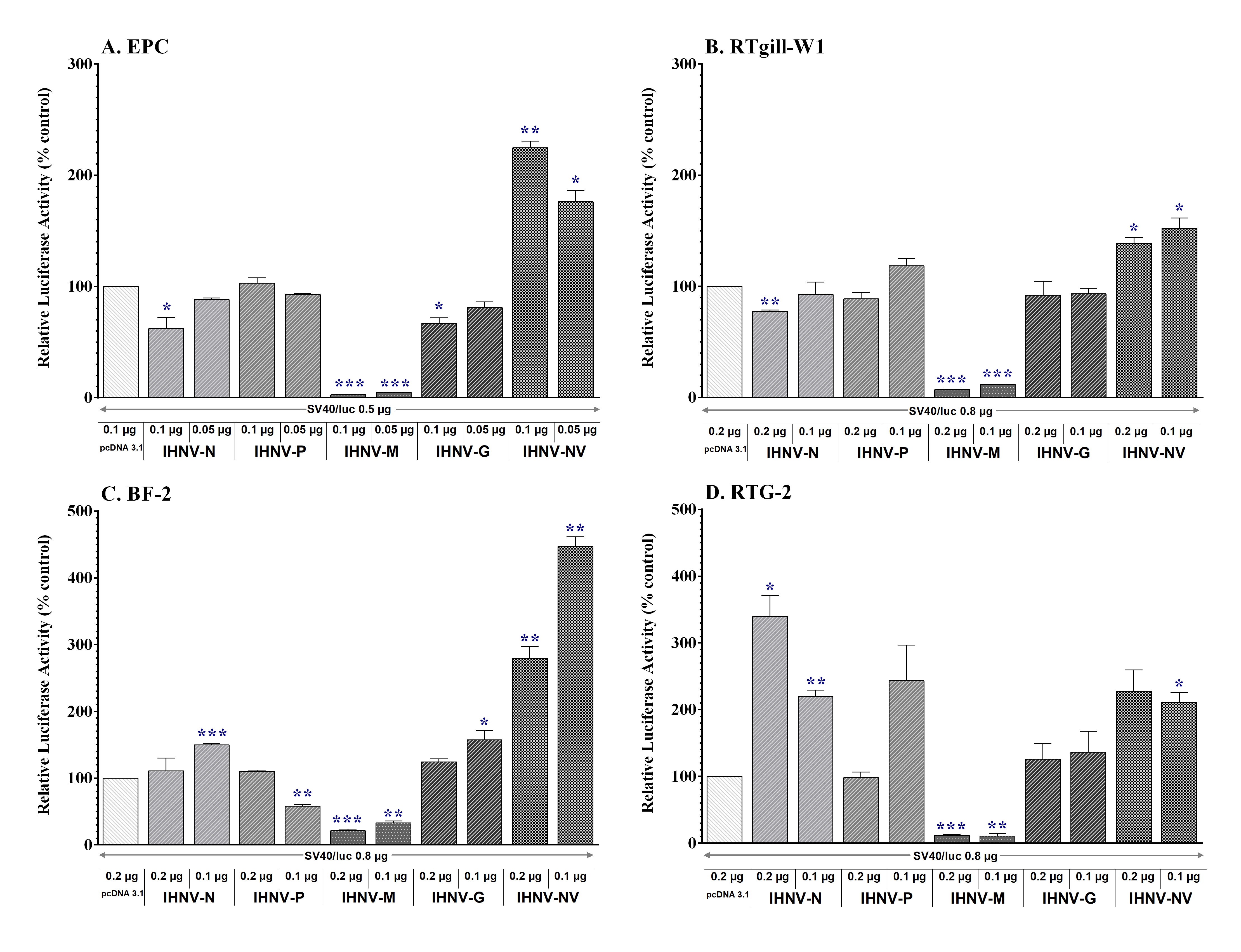

Supplement: Supplementary file 3 — Additional file3: Supp. Fig. 3. Comparative modulation of host constitutive transcription by single IHNV genes. Epithelial (a EPC; b RTgill-W1) and fibroblastic (c BF-2; d RTG-2) cell lines were co-transfected with SV40/luc plus two doses of each IHNV gene plasmid. Closed circular empty pcDNA3.1 plasmid vector was used for transfection balancing and baseline control. Luciferase activity was analyzed at 48 hpt and RLU normalized to total protein concentration in each sample. Data are representative of three independent experiments. Values are group means ±SEM. *p < 0.05; **p < 0.01; ***p < 0.001 indicate significant differences from pcDNA control values as determined by one-way ANOVA and Fisher’s LSD test. [file 12985_2020_1372_MOESM3_ESM.tif]

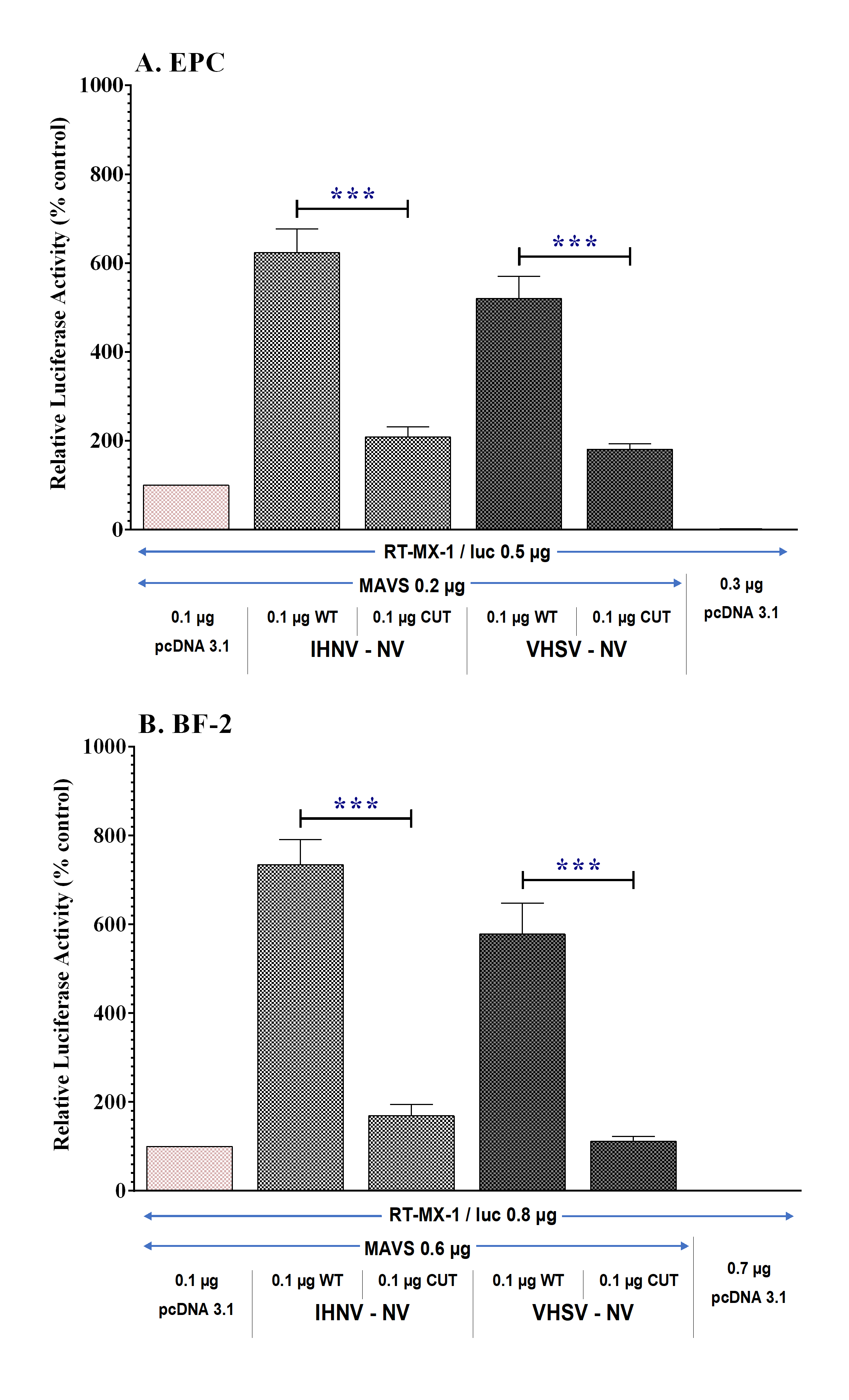

Supplement: Supplementary file 4 — Additional file 4: Supp. Fig. 4. Confirmation of Novirhabdoviruses modulation of the host innate antiviral response operated by NV gene. Epithelial (a EPC) and fibroblastic (b BF-2) cell lines were co-transfected with rainbow trout MX-1/luc, with MAVS as a basal IFN expression stimulator, plus 0.1 μg of intact of destroyed IHNV or VHSV NV gene plasmid. NV plasmids were destroyed upon restriction enzyme cleavage (using Kpn1/EcoRI). Closed circular empty pcDNA3.1 plasmid vector was used for transfection balancing and baseline control. Luciferase activity was analyzed at 72 hpt and RLU normalized to total protein concentration in each sample. Data are representative of three independent experiments. Values are group means ±SEM. *p < 0.05; **p < 0.01; ***p < 0.001 indicate significant differences from pcDNA control values as determined by one-way ANOVA and Fisher’s LSD test. [file 12985_2020_1372_MOESM4_ESM.tif]
